# Serum Cytokeratin 18 Fragment Is an Indicator for Treating Metabolic Dysfunction-Associated Steatotic Liver Disease

**DOI:** 10.1016/j.gastha.2024.08.008

**Published:** 2024-08-14

**Authors:** Miwa Kawanaka, Yoshihiro Kamada, Hirokazu Takahashi, Michihiro Iwaki, Ken Nishino, Wenli Zhao, Yuya Seko, Masato Yoneda, Yoshihito Kubotsu, Hideki Fujii, Yoshio Sumida, Hirofumi Kawamoto, Yoshito Itoh, Atsushi Nakajima, Japan Study Group of NAFLD (JSG-NAFLD), Takeshi Okanoue, Takumi Kawaguchi, Masafumi Ono, Hideyuki Hyogo, Yuichiro Eguchi, Takaomi Kessoku, Hiroshi Ishiba, Miwa Kawanaka, Yoshihiro Kamada, Hirokazu Takahashi, Michihiro Iwaki, Yuya Seko, Hideki Fujii, Yoshio Sumida, Atsushi Nakajima

**Affiliations:** 1 Department of General Internal Medicine 2, Kawasaki General Medical Center, Kawasaki Medical School, Okayama City, Okayama, Japan; 2 Department of Advanced Metabolic Hepatology, Osaka University Graduate School of Medicine, Suita, Osaka, Japan; 3 Faculty of Medicine, Liver Center, Saga University Hospital, Saga University, Saga, Saga, Japan; 4 Faculty of Medicine, Division of Metabolism and Endocrinology, Saga University, Saga, Saga, Japan; 5 Department of Gastroenterology and Hepatology, Yokohama City University School of Medicine Graduate School of Medicine, Yokohama, Kanagawa, Japan; 6 Department of Molecular Gastroenterology and Hepatology, Graduate School of Medicine, Kyoto Prefecture University of Medicine, Kyoto, Kyoto, Japan; 7 Departments of Hepatology, Graduate School of Medicine, Osaka Metropolitan University, Abeno-ku, Osaka, Japan; 8 Graduate School of Healthcare Management, International University of Healthcare and Welfare, Tokyo, Japan

**Keywords:** Cytokeratin 18 Fragment (CK18F), MASH Resolution, Metabolic Dysfunction-Associated Steatotic Liver Disease (MASLD), At Risk MASH, MAFLD Fibrosis Score (MAS score)

## Abstract

**Background and Aims:**

Although numerous noninvasive diagnostic methods have been developed to predict liver fibrosis in metabolic dysfunction-associated steatotic liver disease (MASLD), they lack markers for predicting lobular inflammation, hepatocellular ballooning, or changes related to metabolic dysfunction-associated steatohepatitis (MASH). We examined serum cytokeratin 18 fragment (CK18F) as a noninvasive marker for predicting treatment response and “at-risk MASH” and “MASH resolution” in patients with MASLD.

**Methods:**

One-hundred-and-ten patients with MASLD who underwent repeated biopsy were enrolled (age, 4 [0.5–21] years) in this retrospective study. We investigated associations among serum CK18F levels, liver histology, and blood tests and compared them with changes in serum CK18F levels and liver histology and the resolution of MASH. Additionally, 565 biopsy-proven patients were analyzed for associations among serum CK18F levels, liver histology, and blood tests. Moreover, the Fibrosis-4 (FIB-4) index and CK18F were examined for their usefulness in predicting "at-risk MASH."

**Results:**

CK18F changes were strongly correlated with changes in lobular inflammation, hepatocellular ballooning, and nonalcoholic fatty liver disease activity score. Multiple regression analysis showed that contributing to “MASH resolution” was associated with changes in CK18F levels as independent factors. Patients diagnosed with MASLD and an FIB-4 index >2.67, or those with an FIB-4 index ≤2.67 and CK18F > 200 U/L, were at high risk of developing MASH and should be referred to a hepatologist. Conversely, those with an FIB-4 index ≤2.67 and CK18F ≤ 200 U/L were effectively managed through regular follow-up appointments.

**Conclusion:**

CK18F changes are associated with nonalcoholic fatty liver disease activity score changes and are a promising noninvasive diagnostic marker for "at risk MASH" and "MASH resolution."

## Graphical abstract

**Figure:**
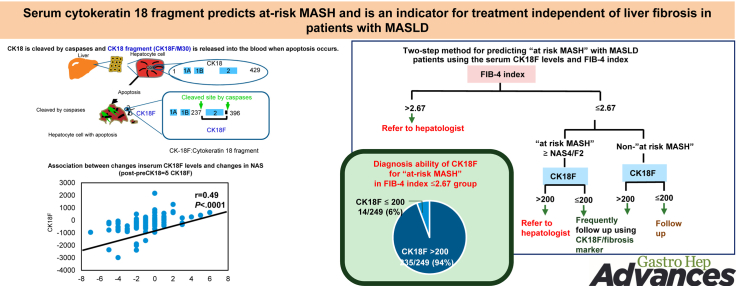

## Introduction

The prevalence of metabolic dysfunction-associated steatotic liver disease (MASLD), including metabolic dysfunction-associated steatohepatitis (MASH), is predicted to surge from 83.1 million in 2015 to 190 million by 2030.^1^, ^2^, ^3^, ^4^, ^5^ MASLD can lead to cirrhosis or hepatocellular carcinoma due to factors, such as insulin resistance, microbiome composition, intestinal flora, and inflammatory cytokines.^4^^,^^6^

MASLD prognosis hinges on liver fibrosis, emphasizing the importance to detect it in patients with advanced fibrosis.^7^^,^^8^ Biomarkers have emerged as noninvasive tools for predicting advanced fibrosis in MASLD.^9^, ^10^, ^11^ Liver fibrosis and steatosis can be quantified using vibration-controlled transient elastography (VCTE) and magnetic resonance elastography (MRE).^12^, ^13^, ^14^ However, the availability of biomarkers that accurately reflect lobular inflammation or hepatocellular ballooning in the liver remains limited,^15^ and only few biomarkers are available to assess treatment effectiveness in MASH. Furthermore, while most liver fibrosis markers effectively diagnose advanced-stage MASH, particularly stages >2–3,^12^ they rarely capture variations in liver fibrosis within stages 0–2, diminishing their usefulness during these stages. Improving the fibrosis stage requires mitigating liver inflammation and steatosis,^16^ and biomarkers reflecting changes in inflammation and steatosis are key indicators of MASH improvement.

Cytokeratins, with a 7–11-nm diameter, constitute the epithelial cell cytoskeleton, comprising approximately 20 different protein types categorized into types I and II based on their different isoelectric points and small molecular weights. Cytokeratin 18 (CK18), an acidic keratin weighing 45 kDa, typically coexists with cytokeratin 18 fragment (CK18F), a cleaved form detectable in the blood during apoptosis.^17^ CK18F is linked to programmed cell death and hepatocyte death and damage.^17^ In MASH, apoptosis is associated with pathogenesis, resulting in elevated blood levels of CK18F.^18^^,^^19^ Although previous studies and meta-analyses have explored CK18F’s utility in distinguishing MASL from MASH,^10^, ^11^, ^12^, ^13^, ^14^, ^15^, ^16^, ^17^, ^18^, ^19^, ^20^, ^21^, ^22^, ^23^ differentiation criteria remain controversial,^24^ particularly concerning burned-out MASH.

Therefore, we examined the utility of the serum CK18F levels in identifying patients with MASLD requiring treatment, termed "at risk MASH," and in predicting treatment efficacy, specifically "MASH resolution."

## Methods

### Patients

We retrospectively identified 110 patients with MASLD who underwent repeated liver biopsy at the Kawasaki Medical School General Medical Center (Okayama, Japan) (n = 95) and Saga University Hospital (Saga, Japan) (n = 15) between 2001 and 2022. Clinical and histological characteristics of the patients are summarized in Table 1 and Table A1.

**Table 1:** Biomarkers and Liver Histology in Patients With MASLD Who Underwent Repeated Liver Biopsies (n = 110)

Parameter	First time	Second time	*P* value
Age, years	55 (19–74)	60 (21–80)	.0051
Diabetes, yes	39%	50%	.1424
Hypertension, yes	42%	45%	.01695
Dyslipidemia, yes	84%	90%	.0690
ALT (IU/L)	78 (14–246)	38 (9–229)	<.0001
AST (IU/L)	55 (14–204)	31 (14–126)	<.0001
γ-GTP (IU/L)	60 (18–751)	35 (12–410)	<.0001
Total cholesterol (ng/dL)	205 (114–347)	191 (121–264)	.0309
Platelet counts (10^4^/ug)	19.8 (10.4–39.4)	19.4 (8.3–40.8)	.2164
Albumin (g/dL)	4.5 (3.2–5.1)	4.3 (2.8–5.1)	.0213
HbA1c (%)	5.6 (4.4–7.1)	5.7 (4.7–13.7)	.3834
Ferritin (ng/mL)	214 (7–950)	108 (1.7–1188)	<.0001
Hyaluronic acid (ng/mL)	35 (9–283)	35 (10–379)	.3283
P-Ⅲ-P (U/mL)	12.8 (6.8–23)	9.59 (3.93–23.1)	.0178
FIB-4 index	1.6 (0.38–5.3)	1.6 (0.29–5.5)	.8293
CK18F (U/L)	570 (114–3204)	32.6 (77–2663)	<.0001

γ-GTP, γ-ghutamyl transpeptidase.

Serum CK18F levels were evaluated in 565 patients with MASLD who underwent liver biopsy at the Kawasaki Medical School General Medical Center (n = 310), Saga University Hospital (n = 162), Yokohama University Hospital (Kanagawa, Japan) (n = 83), and Kyoto Prefectural University (Kyoto, Japan) (n = 10) between 2001 and 2022 (Table 2).

**Table 2:** Clinical Characteristics and Histology of Patients With MASLD-Cross Sectional Study (n = 565)

Parameter	Median
Age, years	58 (18–85)
Sex (M/F)	208/357
Diabetes, yes	54.80%
Hypertension, yes	46.10%
Dyslipidemia, yes	80.10%
ALT (U/L)	55 (9–467)
AST (U/L)	44 (12–201)
γ-GTP (U/L)	52 (12–751)
Total cholesterol (ng/dL)	192 (108–374)
Platelet counts (10^4^/ug)	19.7 (5.7–44.6)
Albumin (g/dL)	4.2 (2.8–5.1)
HbA1c (%)	5.9 (3.9–12.2)
Ferritin (ng/mL)	164 (0.44–1432)
P-Ⅲ-P (U/mL)	0.7 (−0.27–60.7)
Hyaluronic acid (ng/mL)	40 (9–993)
FIB-4 index	1.7 (0.2–12.2)
CK18F (U/L)	398 (39.9–4349)

Factor	Number (n)	(%)
Fibrosis stage		
0	49	8%
1	141	25%
2	142	25%
3	198	35%
4	35	7%
Lobular inflammation		
0	20	4%
1	292	52%
2	212	37%
3	41	7%
Steatosis		
0 (<5%)	13	2%
1 (5%–33%)	269	48%
2 (33%–66%)	214	38%
3 (>66%)	69	12%
Hepatocellular ballooning		
0 (none)	148	26%
1 (few)	276	49%
2 (many)	141	25%
NAS		
0–2	92	16%
3–4	249	44%
5–8	224	40%
0–3	212	38%
4–8	353	62%

γ-GTP, γ-ghutamyl transpeptidase; ALT, alanine aminotransferase.

The study protocol complied with the tenets of the Helsinki Declaration and received approval from the Institutional Review Board of Kawasaki Medical School (No. 6046), which integrated ethical reviews from all participating institutions. All study participants provided informed consent.

### Procedures

We examined the association between changes in CK18F and changes liver histology (liver fibrosis stage, lobular inflammation, steatosis, hepatocellular ballooning, and nonalcoholic fatty liver disease activity score [NAS]) (mean observation period, 4 [0.5–21] years) in 110 patients with MASLD who underwent repeated liver biopsy. Changes in CK18F (δ CK18F) were calculated as post-CK18F minus pre-CK18F (δ CK18F). Change in liver fibrosis, lobular inflammation, steatosis, NAS, and hepatocellular ballooning was defined as a change of at least one stage.

Moreover, individuals whose CK18F levels decreased by 30% between the first and second liver biopsies were defined as improved, while the remainder were classified as nonimproved. Subsequently, these groups were compared in terms of changes observed in the liver tissue parameters (liver fibrosis stage, lobular inflammation, steatosis, hepatocellular ballooning, NAS). The comparison between MASH resolution against other scenarios involved utilizing changes in δCK18F. MASH resolution was defined as an improvement in NAS (defined as a decrease of ≥2 points from baseline to second biopsy in the NAS, which represents the sum of the scores for steatosis [range, 0–3], ballooning [range, 0–2], and lobular inflammation [range, 0–3], with higher scores indicating greater disease activity) and no worsening of fibrosis.^25^^,^^26^

In 565 patients with MASLD undergoing liver biopsy, the serum CK18F levels and Fibrosis-4 (FIB-4) index were evaluated as biomarkers to predict NAS ≥4 and stage ≥2, crucial criteria used to define “at-risk MASH,” and hold significance in MASH clinical trials approved by the Food and Drug Administration.^27^ The CK18F levels cutoff was 200U/L according to these reports.^18^^,^^28^

The MASLD criteria^29^, ^30^, ^31^ include steatotic liver disease and the absence of other liver diseases, individuals with alcohol consumption history (male: <30 g/day, female: <20 g/day) and those meeting at least one of the 5 heart disease risk factors were considered: 1) body mass index ≥23 kg/m^2^ or waist circumference (≥94 cm for men, ≥80 cm for women); 2) elevated fasting blood sugar (≥100 mg/dL), 2-h postprandial blood sugar levels ≥140 mg/dL, HbA1c ≥ 5.7%, and type 2 diabetes or use of antidiabetic drugs; 3) high blood pressure (≥135/85 mmHg) or use of antihypertensive drugs; 4) elevated triglyceride levels (≥150 mg/dL) or use of lipid-improving drugs; and 5) low high-density lipoprotein cholesterol levels (≤40 mg/dL for men and ≤50 mg/dL for women). The FIB-4 index was calculated as aspartate aminotransferase (AST) (IU/L) × age (years)/platelet count (10^9^/L) × AST (IU/L).^32^

### Liver Biopsy and Histological Analysis

All liver biopsies were ultrasound-guided and performed using 16-G or 17-G biopsy needles or laparoscopy-guided using 14-G needles. After diastase digestion, the specimens were fixed in 10% formalin and cut into sections; subsequently, staining with hematoxylin-eosin, Azan, and silver was performed. MASLD diagnosis was confirmed, and the MASLD stage, grade of lobular inflammation, steatosis, and hepatocellular ballooning of the liver tissues were determined according to the Brunt and Kleiner classification.^33^^,^^34^ Histological parameters included fibrosis, inflammation, steatosis, hepatocyte ballooning, and the NAS system. The NAS system, proposed by the MASH Clinical Research Network, evaluated lobular inflammation (0–3), steatosis (0–3), and hepatocellular ballooning (0–2).^33^^,^^34^ Liver fibrosis stages were evaluated according to the Brunt criteria in consultation with 2 experienced pathologist and hepatologist who were blinded to the patients’ clinical and laboratory data.

### Measurement of CK18 Fragment

CK18F levels in the stored samples collected from each hospital were measured using a CK18F enzyme-linked immunosorbent assay kit (TP002; Institute of Immunology, Tokyo, Japan), following the manufacturers’ instructions. The solid-phase sandwich enzyme-linked immunosorbent assay was performed using an anti-CK18 mAb and a horseradish peroxidase-conjugated M30 antibody that specifically binds to CK18F.

### Statistical Analysis

We calculated the median, 25th, and 75th percentiles for continuous factors. Data were analyzed using the Mann–Whitney *U* test for comparisons between 2 groups and the Kruskal–Wallis or chi-square test for comparisons involving 3 or more groups. Associations between CK18F and clinical parameters, as well as liver histology, were determined using Spearman rank sum test, while changes in δCK18F and liver histology were analyzed similarly. Multiple regression analysis was conducted to evaluate the relationship between CK18F and pathological parameters, and MASH resolution was assessed using multivariate logistic regression analysis.

For each continuous variable, optimal cutoff values for sensitivity and specificity were determined using receiver operating characteristic curve analysis. The level of significance was set at *P* < .05. All statistical analyses were performed using JMP software version 16.2 (SAS Institute Inc, Cary, NC, USA).

## Results

### Changes in Serum CK18F Levels Liver Histology in Patients with MASLD Who Underwent Repeated Biopsies

**Figure A1:**
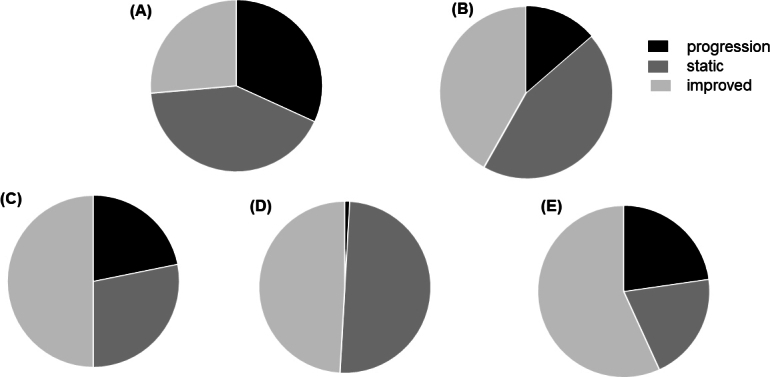
Histological changes in the 110 patients who underwent repeated liver biopsies (mean observation period of 4 [0.5–21] years). Fibrosis stage - improvement: 26 cases; unchanged: 49 cases; progress: 35 cases. Ballooning-improvement: 46 cases; unchanged: 49 cases; progress: 15 cases. Lobular inflammation-improvement: 55 cases; unchanged: 54 cases; progress: one case. Steatosis-improvement: 36 cases; unchanged: 60 cases; progress: 14 cases. NAS- improvement: 63 cases; unchanged: 22 cases; progress: 25 cases (A) Fibrosis stage, (B) lobular inflammation, (C) steatosis, (D) hepatocellular ballooning, and (E) NAS are presented.

Histological changes in the 110 patients who underwent repeat liver biopsy revealed changes in fibrosis (improvement: 26 cases; unchanged: 49 cases; progress: 35 cases), hepatocellular ballooning (improvement: 46 cases; unchanged: 49 cases; progress: 15 cases), lobular inflammation (improvement: 55 cases; unchanged: 54 cases; progress: one case), steatosis (improvement: 36 cases; unchanged: 60 cases; progress: 14 cases), and NAS (improvement: 63 cases; unchanged: 22 cases; progress: 25 cases) (Figure A1).

**Figure 1:**
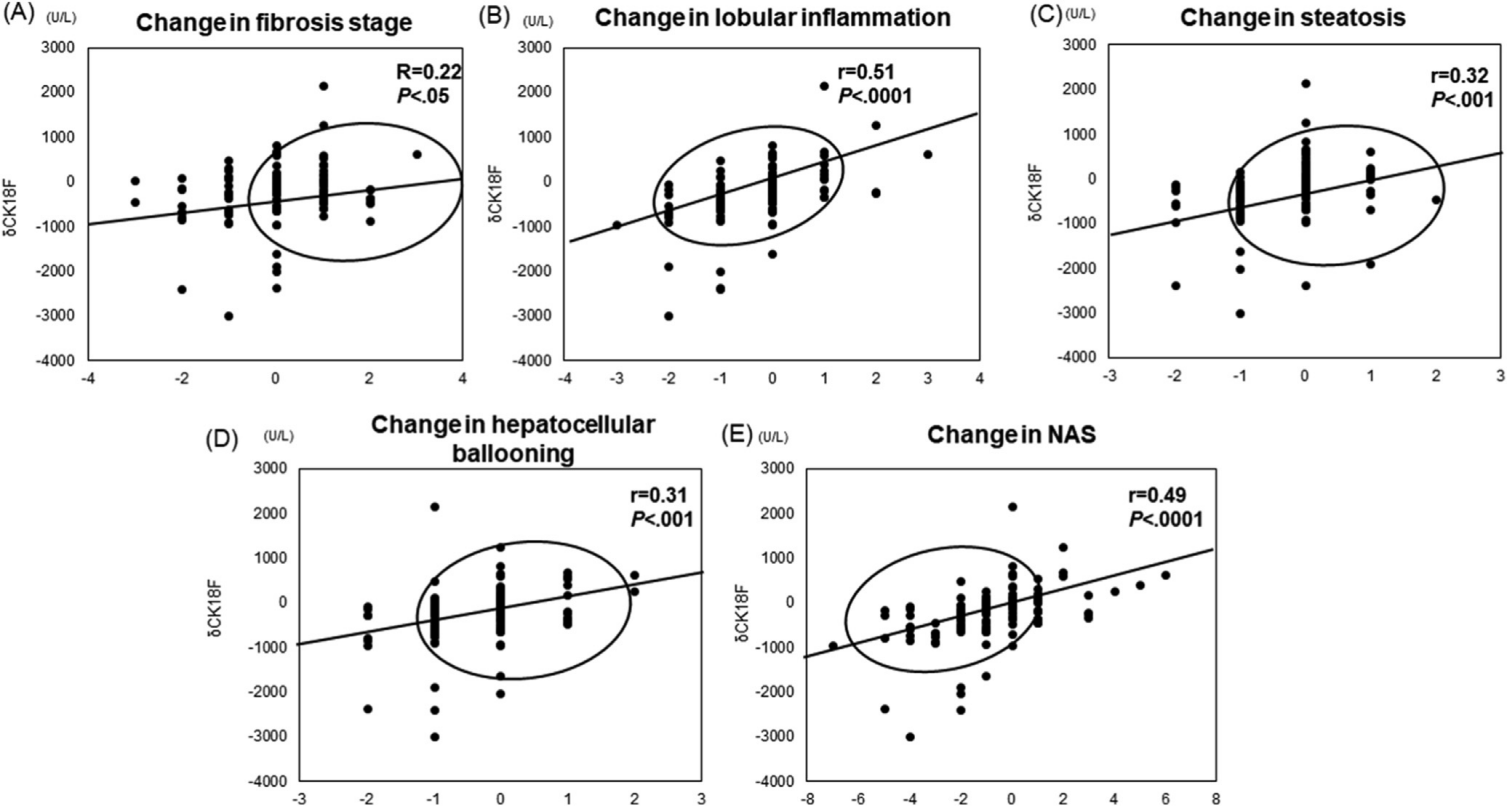
Changes in serum CK18F levels in patients with MASLD who underwent repeated biopsies (post-preCK18 = δ CK18F). (A) Fibrosis stage, (B) lobular inflammation, (C) steatosis, (D) hepatocellular ballooning, (E) NAS. Changes in CK18F (δ value) were strongly correlated with changes in steatosis (r = 0.32, *P* < .001), hepatocellular ballooning (r = 0.31, *P* < .0001), lobular inflammation (r = 0.51, *P* < .0001), and NAS (r = 0.49, *P* < .0001). There was a weak correlation between changes in CK18F and fibrosis (r = 0.22, *P* < .05).

When examining changes in CK18F (δ value) alongside alterations in liver histology, significant correlations were observed with changes in steatosis (r = 0.32, *P* < .001), hepatocellular ballooning (r = 0.31, *P* < .0001), lobular inflammation (r = 0.51, *P* < .0001), and NAS (r = 0.49, *P* < .0001). However, a weaker correlation was noted between changes in CK18F and fibrosis (r = 0.22, *P* < .05; Figure 1).

**Figure 2:**
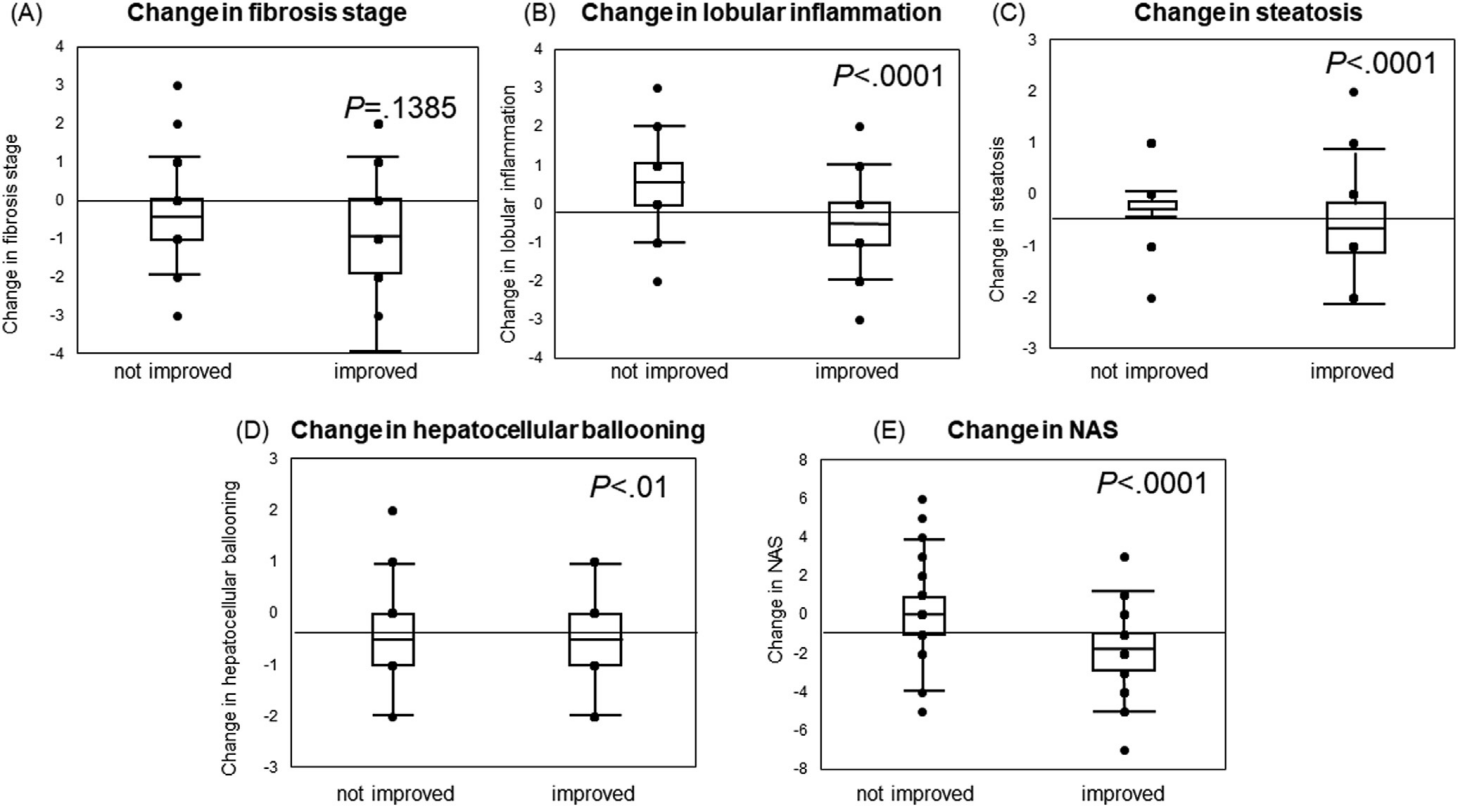
Changes in serum CK18F levels and liver histology in patients with MASLD who underwent repeated biopsies. A 30% decrease in CK18F in the baseline and second liver biopsies indicated improvement, while any other change was categorized as nonimproved. (A) Fibrosis stage, (B) lobular inflammation, (C) steatosis, (D) hepatocellular ballooning, and (E) NAS are presented. Patients who demonstrated 30% improvement in CK18F exhibited significantly improved steatosis (*P* < .0001), hepatocellular ballooning (*P* < .01), and NAS (*P* < .0001), with no difference in fibrosis stage.

Furthermore, those who exhibited a 30% decrease in CK18F between the baseline and second liver biopsies were defined as improved, while the remaining cases were categorized as nonimproved. Upon comparison with changes in liver tissue, improved cases showed enhancements in lobular inflammation compared to nonimproved cases. Additionally, improvements were observed in NAS (*P* < .0001), steatosis (*P* < .0001), hepatocellular ballooning (*P* < .01), while no significant difference was noted in fibrosis stage (Figure 2).

**Figure 3:**
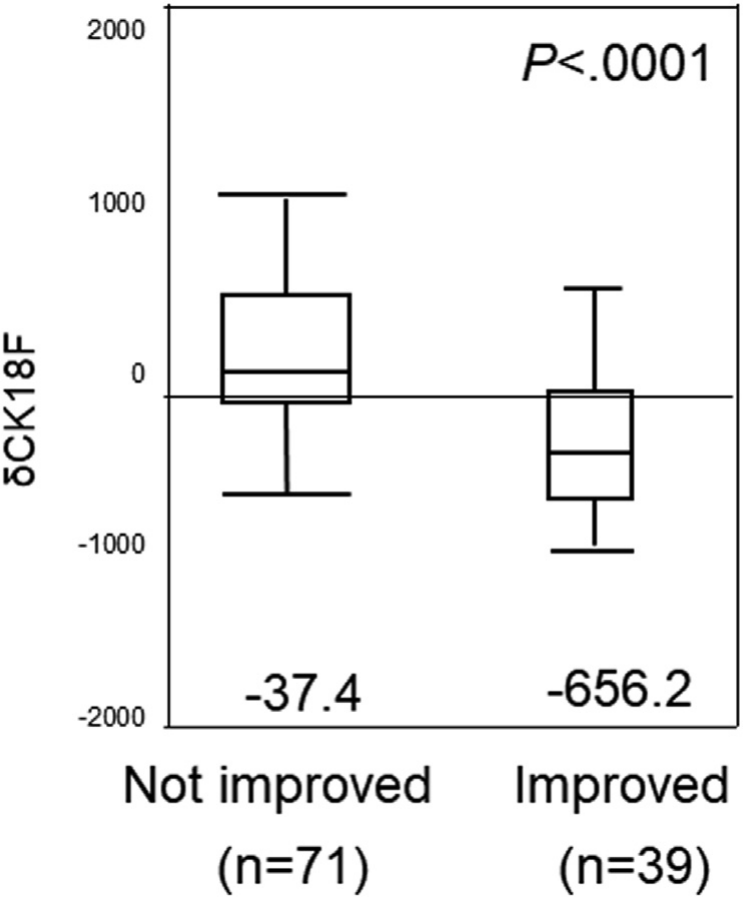
Changes in the serum CK18F levels in patients with MASLD who underwent repeated biopsies (post−pre CK18F = δ CK18F). δCK18F was an independent factor for MASH resolution in multivariate logistic regression analysis.

A significant difference in δ CK18F was noted between patients who experienced MASH resolution without worsening liver fibrosis (n = 39) and those who did not (n = 71) (*P* < .0001). In multivariate logistic regression analysis assessing factors contributing to MASH resolution, δ CK18F emerged as an independent factor for MASH resolution (Figure 3).

### Prediction of “At-risk MASH” Using Markers of Serum CK18F Levels and Liver Fibrosis Markers

**Figure 4:**
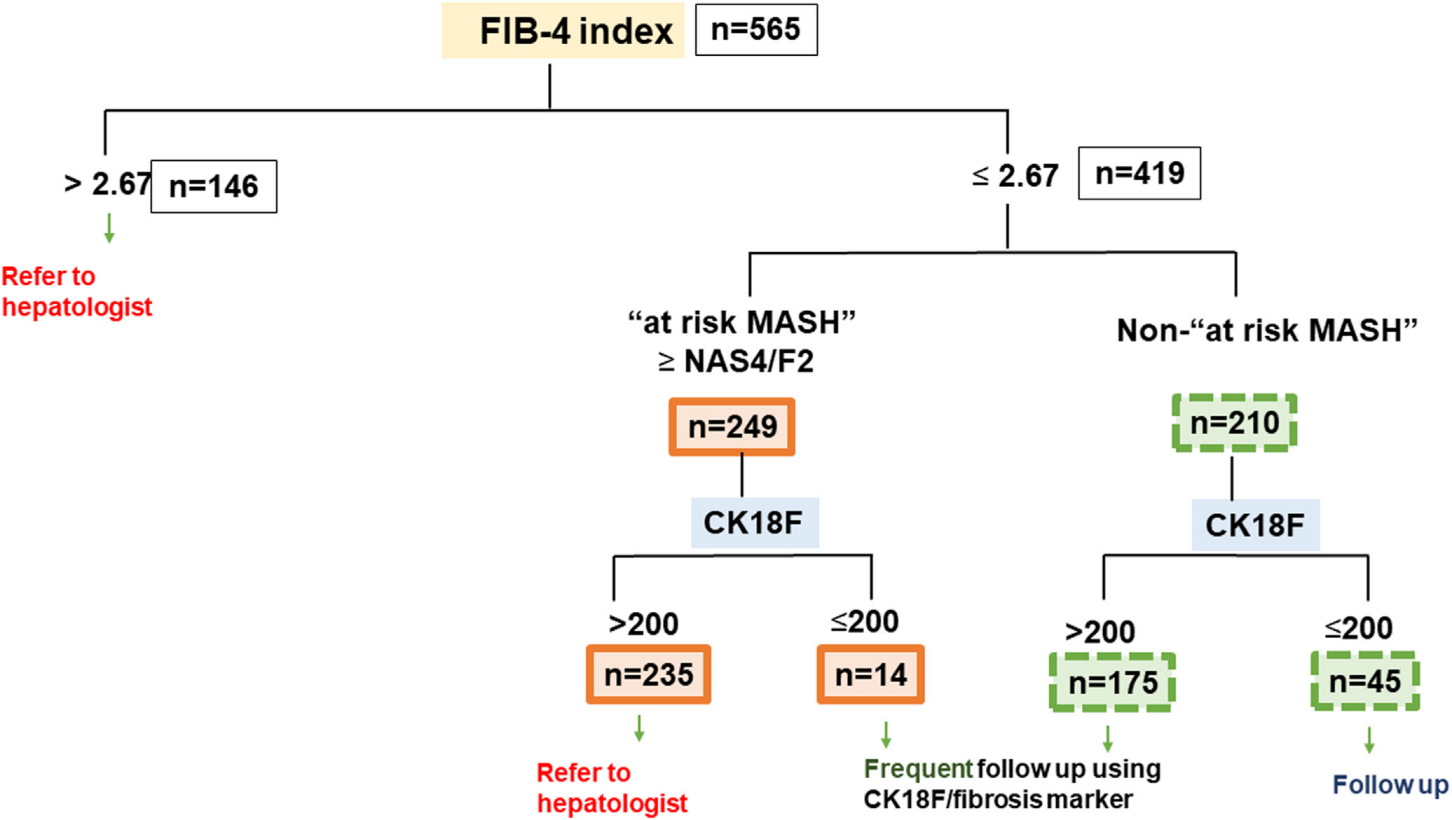
Patients with MASLD and an FIB-4 index >2.67 (146 patients) should be referred to a hepatologist. Of 249 patients with an FIB-4 index ≤2.67 and CK18F > 200 U/L, 235 (94%) were highly likely to have “at-risk MASH” and had to be referred to a hepatologist. Among patients with MASLD with an FIB-4 index ≤2.67 and CK18F ≤ 200 U/L, 45 (76.2%) of 59 patients could be ruled out of “at-risk MASH” and be followed up with routine monitoring.

Patients with MASLD and an FIB-4 index >2.67 should be referred to a hepatologist, as they are more likely to have advanced liver fibrosis. Even if the FIB-4 index is ≤ 2.67, patients with a CK18F value > 200 U/L are susceptible of "at-risk MASH" and require evaluation by a hepatologist. MASLD patients with an FIB-4 index ≤2.67 and CK18F ≤ 200 U/L can be excluded from "at-risk MASH" and may be monitored through routine follow-up (Figure 4).

## Discussion

Serum cytokeratin-18 fragments have been most extensively evaluated, with a pooled sensitivity of 66% and specificity of 82% for the diagnosis of NASH.^35^^,^^36^ However, previous meta-analyses have been shown to be inadequate for the diagnosis of MASH, and a two-step approach using CK-18 and FGF21 or FIB4-Index has been reported to further improve the accuracy of the diagnosis of MASH.^20^^,^^25^^,^^37^^,^^38^

Our study highlights the serum CK18F level as a biomarker associated with liver tissue inflammation and hepatocellular ballooning, particularly in NAS. While numerous noninvasive markers have been proposed for advanced liver fibrosis, reports on biomarkers reflecting lobular inflammation, steatosis, and hepatocyte ballooning stages preceding fibrotic changes remain limited. Existing markers of liver fibrosis fail to reflect the treatment efficacy, particularly in cases where fibrosis is at stage ≥3.^9^^,^^32^^,^^39^ Consequently, transitions from stage 0 to 2 or from stage 2 to 0 are often undetected by these markers. However, serum CK18F levels exhibit significant improvements.^11^

Moreover, regarding MASH, liver fibrosis evolves over several years, rendering fibrosis markers ineffective as short-term indicators of treatment efficacy within 1–2 years.^40^ However, the serum CK18F levels are promising markers of inflammation, steatosis, and ballooning, which are early changes and can serve as useful indicators of treatment response. Currently, liver biopsies are performed as short-term treatment efficacy indicators, but performing liver biopsies poses challenges owing to potential sampling errors and variations in physician interpretations.

MRE, magnetic resonance imaging (MRI)- equiring treatment for "MASH resolution.”^26^ Conversely, predicting at-risk MASLD is equally important.

In patients with MASLD, an FIB-4 index >2.67 warrants consultation with a hepatologist. However, for those with an FIB-4 index ≤2.67, the clinical decision is challenging. In our study, 94% of patients with FIB-4 ≤2.67 but CK18F > 200 U/L were predicted to be "at risk for MASH." Combining the FIB-4 index and CK18derived proton density fat fraction, and VCTE are simple methods to assess liver hardness and fat content.^13^^,^^14^^,^^41^ However, these methods require specific equipment and do not provide insights into inflammation. Given the lack of biomarkers for liver injury, serum CK18F levels emerge as potentially useful as a potentially valuable biomarker to reflect NAS changes and “MASH resolution.”

Furthermore, our findings suggest that utilizing liver fibrosis markers, such as the FIB-4 index in combination with CK18F, may contribute to identifying MASLD cases rF levels allows prediction of liver fibrosis, steatosis, lobular inflammation, and hepatocellular ballooning, providing valuable insights for future treatment strategies and complication detection.

This study is the first to predict biomarker-based MASH risk by combining FIB-4 index and CK18F levels and to predict tissue changes due to inflammatory changes preceding fibrotic changes. The utility of MRI-based scores (MAST score),^42^ Fibroscan-AST score,^43^ and MRE plus FIB-4^44^ in predicting MASH risk has been reported. However, measuring these is difficult without VCTE or MRE systems. Predicting MASH risk using only blood tests has the advantage of being easily performed at any facility.

Moreover, CK18F can assess improvement in NAS without fibrosis progression (Figure 3), commonly used in evaluating global clinical trials,^4^^,^^26^ and suggests the potential to replace liver biopsy in assessing treatment response.

As mentioned above, previous reports have indicated that CK18F inadequately evaluated patients with MASH, possibly because of its utility in discriminating MASH from MASL.^20^^,^^23^ Meta-analyses have demonstrated limited utility in MASH diagnosis,^24^ mainly because CK18F correlates with ballooning and lobular inflammation but not with fibrosis. Therefore, in MASH cirrhosis, CK18F levels decrease due to reduced steatosis, lobular inflammation, and NAS (termed "burned-out" MASH).^18^^,^^34^ Consistent with previous findings, our study showed lower CK18F levels in fibrosis stage 4 compared to stage.^38^

**Figure A2:**
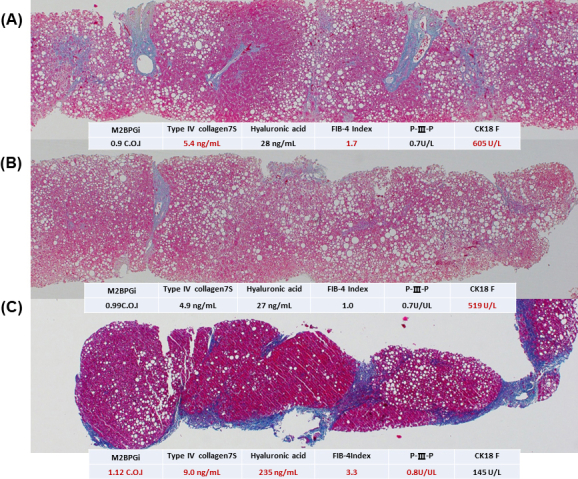
Cirrhosis progressed and burned out, CK18F levels decreased despite the increase in liver fibrosis markers. (A) First biopsy, (B) second biopsy (first biopsy after 2 years), (C) third biopsy (first biopsy after 13 years). At 13 years after the first liver biopsy, liver fibrosis progressed and liver fibrosis markers increased, but CK18F decreased.

**Figure A3:**
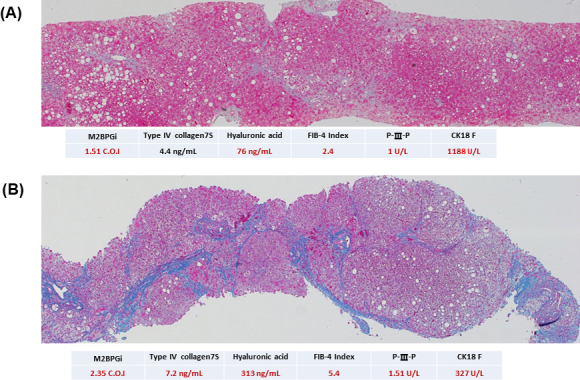
(A) First biopsy and (B) second biopsy (first biopsy after 10 years). At 10 years after the initial liver biopsy, liver fibrosis progressed to burned-out MASH. Despite elevated liver fibrosis markers, CK18F levels decreased.

Herein, we observed a decrease in the CK18F levels as cirrhosis progressed to a burned-out state, despite increasing liver fibrosis markers (Figures A2 and A3). This phenomenon complicates the discrimination between MASH and MASL based solely on serum CK18F levels. However, CK18F has proven usefulness in diagnosing MASH in MASLD with low fibrosis. In previous reports,^28^^,^^38^ CK18F has demonstrated utility in diagnosing MASH in MASLD cases with an FIB-4 index ≤2.67. Serum CK18F levels can differentiate cases with advanced fibrosis and concomitant lobular inflammation and steatosis from those without inflammation and steatosis (including burned-out MASH), and cases with advanced fibrosis but decreased inflammation and steatosis due to treatment. Combining CK18F and fibrosis markers may aid in diagnosing burned-out MASH, particularly since MASLD is often undetected during progression due to the gradual decline in platelet and albumin levels with advanced fibrosis.^45^

Another reason that initially impeded the consideration of CK18F as a biomarker for MASLD is its susceptibility to degradation during frozen storage, making quality control of stored sera difficult and its utility debatable. In Japan, this issue was resolved by storing at −80 °C and developing a rapid assay (1.5 h), leading to its approval.^46^

Recent studies have used the CK18F levels as indicators of successful treatment in MAFLD complicated with type 2 diabetes mellitus, such as with duodenal-jejunal bypass and Mediterranean diets.^47^ However, these studies did not compare CK18F values with those of actual liver tissue or specify the particular part of the liver tissue that improved. The FALCON1 trial^48^ assessed histological changes (liver fibrosis, lobular inflammation, and steatosis) and changes in biomarkers, MRE, and MRI-derived proton density fat fraction after 12 and 24 weeks of pegbelfermin treatment. Serum CK18F levels strongly correlated with lobular inflammation, ballooning, and the pro-peptide of type III collagen in MASH.^48^ Furthermore, decrease in CK18 was observed in Resmetiron, the first drug in the world to be approved as a treatment for MASH, suggesting its potential as a marker of therapeutic efficacy.^49^

Previously, we reported the association of serum CK18F levels with liver histology and NAS; however, these were single-center studies, although the results were similar to those of the present multicenter study. Additionally, this multicenter study did not assess changes in CK18 levels.^38^

In summary, CK18F levels were correlated with lobular inflammation, hepatocellular ballooning, and NAS, reflecting tissue changes irrespective of fibrosis. Combining the FIB-4 index and CK18F levels can assist in confirming or ruling out MASH cases requiring treatment. Additionally, it holds promise as a noninvasive diagnostic marker, independent of fibrosis, to predict disease progression and improvement at an early stage, suggesting its potential in advancing MASH treatment.

## Supplementary Materials

Table A1
