# Supplementary material for: Serum Cytokeratin 18 Fragment Is an Indicator for Treating Metabolic Dysfunction-Associated Steatotic Liver Disease
Source: Gastro Hep Adv. 2024 Aug 14;3(8):1120–8. doi: 10.1016/j.gastha.2024.08.008 (PMC11554606; doi:10.1016/j.gastha.2024.08.008)
Supplement: Table A1 [file mmc1.docx]

**Supplementary Table 1S** Characteristics liver histology of patients with NAFLD who underwent repeated liver biopsies

| Stage | 0 | 10 | 9% |
| --- | --- | --- | --- |
|  | 1 | 28 | 25% |
|  | 2 | 27 | 25% |
|  | 3 | 45 | 41% |
|  | 4 | 0 | 7% |
| Lobular Inflammation | 0 | 5 | 5% |
|  | 1 | 40 | 36% |
|  | 2 | 46 | 42% |
|  | 3 | 19 | 17% |
| Steatosis | 0 (<5%) | 0 | 0% |
|  | 1 (5–33%) | 40 | 36.5% |
|  | 2 (33–66%) | 52 | 47% |
|  | 3 (>66%) | 18 | 16.5% |
| Hepatocellular ballooning | 0 (None) | 18 | 16% |
|  | 1 (Few) | 48 | 44% |
|  | 2 (Many) | 44 | 40% |
| NAS | 0–2 | 11 | 10% |
|  | 3–4 | 40 | 36% |
|  | 5–8 | 59 | 54% |
|  | 0–3 | 28 | 25% |
|  | 4–8 | 82 | 75% |
